# ﻿Exploring cryptic biodiversity in a world heritage site: a new pitviper (Squamata, Viperidae, Crotalinae) from Jiuzhaigou, Aba, Sichuan, China

**DOI:** 10.3897/zookeys.1114.79709

**Published:** 2022-07-25

**Authors:** Mei-Hua Zhang, Sheng-Chao Shi, Cheng Li, Peng Yan, Ping Wang, Li Ding, Jie Du, Anđelka Plenković-Moraj, Jian-Ping Jiang, Jing-Song Shi

**Affiliations:** 1 China-Croatia “Belt and Road” Joint Laboratory on Biodiversity and Ecosystem Services, Chengdu Institute of Biology, Chinese Academy of Sciences, Chengdu 610041, China Chengdu Institute of Biology, Chinese Academy of Sciences Chengdu China; 2 Institute of Zoology, Chinese Academy of Sciences, Beijing 100101, China Shenyang Normal University Shenyang China; 3 Key Laboratory of Vertebrate Evolution and Human Origins, Institute of Vertebrate Paleontology and Paleoanthropology, Chinese Academy of Sciences, Beijing 100044, China Jiuzhaigou Nature Reserve Administrative Bureau Zhangzha China; 4 Colleges of Life Sciences, Shenyang Normal University, Shenyang 110000, China University of Zagreb Zagreb Croatia; 5 Jiuzhaigou Nature Reserve Administrative Bureau, Zhangzha Town, Jiuzhaigou 623402, China Institute of Zoology, Chinese Academy of Sciences Beijing China; 6 Department of Biology, Faculty of Science, University of Zagreb, Zagreb 10000, Croatia Institute of Vertebrate Paleontology and Paleoanthropology, Chinese Academy of Sciences Beijing China

**Keywords:** Asian pitviper, *Gloydiuslateralis*, Jiuzhaigou National Nature Reserve, morphology, new species, phylogenetics

## Abstract

This study presents a comprehensive morphological comparison along with molecular phylogeny of the genus *Gloydius* based on five mitochondrial genes (12S, 16S, COI, cytb, and ND4). The specimens collected from Jiuzhaigou National Nature Reserve are shown to be a new species, *Gloydiuslateralis***sp. nov.** Zhang, Shi, Jiang & Shi based on a combination of morphological and molecular accounts. *G.lateralis***sp. nov.** differs from other congeneric species by a series of diagnostic morphological characteristics and forms a strongly supported monophyletic group. The new species is phylogenetically closely related to *G.swild*, another recently described species from Heishui, Aba, Sichuan.

## ﻿Introduction

Jiuzhaigou National Nature Reserve (JNNR; 32.900–33.266°N, 103.767–104.050°E, 1996–4764 m a.s.l.), a World Heritage Site, lies in the transition zone from the eastern edge of the Qinghai-Tibet Plateau to the Sichuan Basin (Sichuan Province, China), and occupies an area of 651 km^2^ (Li et al. 2004). The reserve is covered with well-preserved original forests, and numerous alpine lakes, inhabiting many world-famous rare animals, such as Giant Panda (*Ailuropodamelanoleuca*), and Golden Snub-nosed Monkey (*Rhinopithecusroxellana*). In contrast to the mammals, the herpetological diversity here is relatively low due to the harsh alpine environment (e.g., low temperatures, low oxygen levels, and intense solar radiation: Li et al. 2004; Shi et al. 2017). To further investigate the herpetological biodiversity and post-earthquake ecological system in this region, we conducted a series of investigations from April to September 2021, and collected several specimens of small-bodied pitvipers in the genus *Gloydius* Hoge & Romano-Hoge, 1981.

Pitvipers of the genus *Gloydius*, or Asian pitvipers, are small-bodied venomous snakes distributed in Asia. At present, at least 23 species of the genus belonging to three groups (i.e., the *G.blomhoffii* group, *G.halys-intermedius* group, and *G.strauchi* group) are recognized (Orlov and Barabanov 1999; Zhao 2006; Shi et al. 2017, 2018). From 2017 to 2021, five taxa initially thought to be *G.strauchi* (Bedriaga, 1912) had been shown to be new species based on morphological and genetic accounts: *Gloydiusrubromaculatus* (Shi et al. 2017), *G.angusticeps* (Shi et al. 2018), *G.huangi* (Wang et al. 2019), *G.lipipengi* (Shi et al. 2021), and *G.swild* (Shi et al. 2021). Previous studies have suggested that there still might be hidden species within the *G.strauchi* complex, given that the *G.strauchi* complex is widely distributed in western China (Zhao et al. 1998; Zhao 2006; Shi et al. 2021). Shi et al. (2018) indicated the underestimated diversity of alpine pitvipers, and emphasized the necessary of further elucidating of the biodiversity in southwest China.

During the herpetological surveys in JNNR up to August 2021, we collected nine specimens of *Gloydius* from Zharu Valley. Subsequent examination of these specimens, and assessment of their morphological and genetic data showed that these individuals differ from the topotypic *G.angusticeps*, *G.strauchi*, and *G.swild* from Sichuan Province, as well other congers of the genus. Herein, we report a new *Gloydius* species. The discovery of this new species once more highlights the species diversity of *Gloydius* in the Hengduan Mountains.

## ﻿Materials and methods

### ﻿Sampling

**Figure 1.**
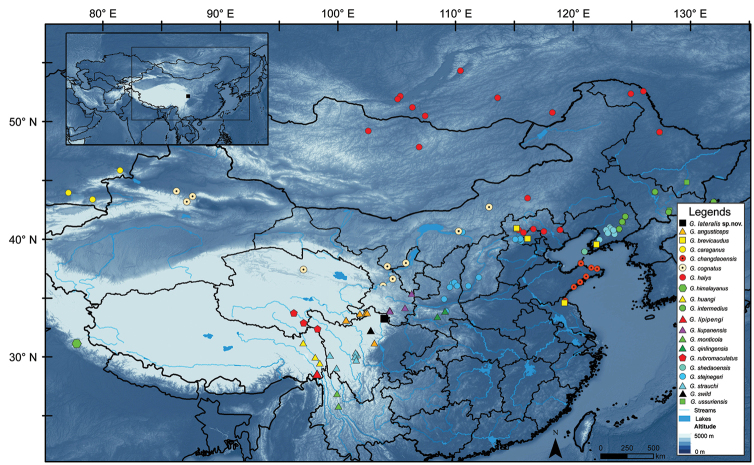
The type locality of *Gloydiuslateralis* sp. nov. in Jiuzhaigou National Nature Reserve, denoted by a black square, with the collection localities of some other congeneric species.

Nine specimens collected from Jiuzhaigou National Nature Reserve were fixed in 10% buffered formalin after removing the liver tissues for molecular analyses, and then transferred to 80% ethanol for permanent preservation (Fig. 1). The above-mentioned specimens were deposited in the Institute of Chengdu Institute of Biology (**CIB**), Chinese Academy of Sciences (**CAS**), Institute of Vertebrate Paleontology and Paleoanthropology (**IVPP**), Chinese Academy of Sciences (**CAS**) and Sichuan Academy of Forestry (**SAFS**).

### ﻿Morphology

A total of 15 morphological characters of the candidate new species were measured. Snout-vent length (**SVL**), tail length (**TL**), and total length (**TTL**) were measured with a measure to the nearest 1 mm. Other morphological measurements were taken with Vernier calipers to the nearest 0.1 mm: head length (**HL**, from the tip of snout to the line connecting left and right posterior margins of mandible), head width (**HW**, the widest part of the head in dorsal view), head depth (**HD**, the deepest part of the head in lateral view), snout length (**SL**, from the tip of snout to the anterior margin of the eye), eye diameter (**ED**, measured as a horizontal distance), interorbital space (**IOS**, the distance between the top margin of eyes), and internasal space (**INS**, the distance between nostrils). The numbers of supralabials (**SPL**), infralabials (**IFL**), dorsal scales (**DS**), ventral scales (**V**, excluding preventral scales), and subcaudal scales (**SC**) were counted. Dimensions and counts are listed in Table 1. Coloration is described according to the HTML color codes (https://html-color.codes). For morphological comparison, specimens of seven congers also from eastern part of Qinghai-Tibet Plateau were examined (Table 1).

**Table 1.** Measurements of *Gloydiuslateralis* sp. nov. and other species of the genus from eastern part of Qinghai-Tibet Plateau.

Taxa	Museum Vouchers	Preserve	Localities	Gender	SVL	TTL	TL	HL	HW	HH	SL	ED	IOS	INS	V	Sc	DS	SPL (L/R)	IFL (L/R)	ED/HL	Reference
*Gloydiuslateralis* sp. nov.	CIB 119377 (JZ02)**	CIB	JNNR	F	440.0	498.0	58.0	21.9	15.1	8.3	6.4	3.2	7.8	4.2	158	38	20-20-15	6/6	10/10	0.146	This study
*G.lateralis* sp. nov.	IVPP OV 2727 (JZ01)*	IVPP	JNNR	F	428.0	434.1	60.1	19.2	14.4	7.4	6.2	3.2	8.2	4.3	161	44	21-21-15	8/7	10/10	0.167	This study
*G.lateralis* sp. nov.	CIB 119378 (JZ03)*	CIB	JNNR	F	421.0	478.0	57.0	18.5	13.4	8.5	6.8	2.9	7.8	5.1	155	39	21-21-16	7/7	10/10	0.157	This study
*G.lateralis* sp. nov.	CIB 119379 (JZ04)*	CIB	JNNR	F	393.0	454.0	61.0	19.3	12.0	6.3	5.3	2.8	7.4	3.8	158	42	21-20-16	7/7	11/11	0.145	This study
*G.lateralis* sp. nov.	CIB 87280*	CIB	JNNR	M	394.0	464.7	70.7	21.0	15.2	8.0	6.4	3.6	8.5	4.3	151	49	21-21-15	7/7	9/10	0.171	This study
*G.lateralis* sp. nov.	SAFS2021001*	SAFS	JNNR	F	445.0	481.0	36+	23.0	1.6	8.2	–	–	9.1	4.0	155	22+	21-21-17	7/7	10/10	–	This study
*G.lateralis* sp. nov.	SAFS2021002*	SAFS	JNNR	F	489.0	533.0	44+	23.0	1.6	8.4	–	–	9.4	4.0	163	22+	21-21-17	7/7	10/10	–	This study
*G.lateralis* sp. nov.	SAFS2021003*	SAFS	JNNR	F	430.0	488.0	58.0	24.0	1.5	7.8	–	–	9.1	4.0	162	41	21-21-17	7/7	10/10	–	This study
*G.lateralis* sp. nov.	SAFS2021004*	SAFS	JNNR	F	376.0	424.0	48+	20.0	1.2	6.5	–	–	7.0	3.3	160	33+	21-21-17	7/7	10/10	–	This study
*G.angusticeps*	IVPPOV 2634**	IVPP	Xiaman, Sichuan	M	373.2	439.7	66.5	21.2	12.4	6.6	6.7	2.2	9.1	4.1	148	39	19-19-15	7/7	10/10	0.104	Shi et al. (2018)
*G.angusticeps*	JS1507G5A*	SYNU	Xiaman, Sichuan	M	283.4	331.6	42.2	16.9	9.8	6.3	4.5	2.0	7.5	3.3	151	39	19-20-15	6/6	9/10	0.118	Shi et al. (2018)
*G.angusticeps*	JS1306G1A*	SYNU	Golog, Qinghai	F	443.1	502.3	59.2	23.6	13.2	7.0	5.3	2.8	8.3	4.3	162	31	21-21-15	7/6	8/9	0.119	Shi et al. (2018)
*G.angusticeps*	IOZ002317*	IOZ	Golog, Qinghai	F	457.2	459.4	72.2	22.1	11.8	7.1	–	–	8.0	4.5	157	35	19-21-15	6/6	10/10	–	Shi et al. (2018)
*G.huangi*	KIZ 027654**	KIZ	Chaya, Chamdo, Tibet	F	532.0	455.0	67.0	23.2	14.6	–	–	3.1	8.4	4.3	174	43	21-21-15	7/7	10/10	0.134	Wang et al. (2019)
*G.lipipengi*	IVPP OV 2720**	IVPP	Zawalong, Zayu, Tibet	M	540.6	628.2	87.6	25.2	13.2	8.2	7.4	2.9	9.6	5.4	165	46	23-21-15	7/7	10/11	0.115	Shi et al. (2021)
*G.monticola*	CIB 72553	CIB	Zhongdian, Yunnan	F	274.0	308.0	34.0	18.1	9.5	6.4	–	1.5	6.9	4.7	145	30	19-19-15	6/6	9/10	0.083	Shi et al. (2017)
*G.rubromaculatus*	IOZ 032317**	IOZ	Yushu, Qinghai	M	473.0	554.0	81.0	24.6	15.8	7.4	7.8	3.1	8.2	4.6	158	43	21-21-15	7/8	10/11	0.126	Shi et al. (2017)
*G.strauchi*	SUNU1410G3△	SYNU	Kangding, Sichuan	M	407.3	482.7	75.4	21.5	13.4	7.8	–	2.8	9.3	4.4	144	45	21-21-15	7/7	10/10	0.130	Shi et al. (2017)
*G.strauchi*	CIB 14356△	CIB	Kangding, Sichuan	M	338.5	405.0	66.3	19.4	11.8	6.2	–	2.1	7.7	4.2	151	38	21-21-16	7/7	–	0.108	Shi et al. (2017)
*G.strauchi*	CIB 14357△	CIB	Kangding, Sichuan	M	347.2	412.4	65.2	19.9	12.1	8.7	–	2.2	7.8	3.7	146	41	21-21-15	7/7	–	0.111	Shi et al. (2017)
*G.strauchi*	SYNU1508G4	SYNU	Litang, Sichuan	M	372.3	436.4	64.1	20.3	12.7	6.5	5.9	2.1	8	4.3	148	42	21-21-15	7/7	10/10	0.103	Shi et al. (2017)
*G.strauchi*	CIB 78588	CIB	Litang, Sichuan	M	427.3	504.6	77.3	24.6	15.6	8.2	–	2.7	9.9	5.3	151	40	21-21-16	7/7	10/10	0.110	Shi et al. (2017)
*G.strauchi*	CIB 14358△	CIB	Kangding, Sichuan	F	384.1	438.3	54.2	22.4	12.4	7.9	–	2.4	8.4	5.6	158	35	21-21-15	7/7	–	0.107	Shi et al. (2017)
*G.strauchi*	CIB 14359△	CIB	Kangding, Sichuan	F	450.3	505.5	55.2	20.9	12.4	7.2	–	1.9	7.8	6	160	33	21-21-15	7/7	–	0.091	Shi et al. (2017)
*G.swild*	IVPP OV 2725**	IVPP	Heishui, Aba, Sichuan	F	462.0	529.5	67.5	20.8	12.2	6.6	5.8	2.4	7.6	4.1	170	46	21-21-15	7/7	10/10	0.115	Shi et al. (2021)
*G.swild*	IVPP OV 2726*	IVPP	Heishui, Aba, Sichuan	F	552.0	629.1	77.1	23.8	15.7	8.4	6.2	3.2	9.6	5.0	168	43	21-21-17	7/7	10/10	0.134	Shi et al. (2021)

Note: **, holotype; *, paratype; △, topotype. The missing data are marked as “–”.

### ﻿Molecular analyses

Genomic DNA was extracted from four specimens collected in this study using the Qiaprep Spin Miniprep kit (QiaGen). Five mitochondrial genome fragments were specifically amplified for this study: an 859 bp fragment of 12S ribosomal RNA (12S) using primers 12SFPhe and 12SRVal (Knight and Mindell 1993); a 465 bp fragment of 16S ribosomal RNA (16S) using primers 16sFL and 16sRH (Palumbi et al. 1991); a 657 bp fragment of cytochrome c oxidase subunit I (COI) using primers L14919 and H16064 (Burbrink et al. 2000); a 1065 bp fragment of cytochrome b (cytb) using primers L14919 and H16064 (Burbrink et al. 2000); and a 666 bp fragment of NADH dehydrogenase subunit 4 (ND4) using the primers ND4 and Leu (Arevalo et al. 1994). The standard PCR protocol was performed in a 20 µl reaction with at least 20 ng of template DNA and 10 *p*mol of primers. PCR conditions consisted of an initial denaturation for 3 min at 94 °C, followed by 35 cycles: denaturation at 94 °C for 30 sec, annealing temperature 52 °C for 12S, 54 °C for 16S, 50 °C for COI, 48 °C for cytb, and 56 °C for ND4 for 30 sec, elongation at 72 °C for 60 sec, and then finalized with an extension step at 72 °C for 10 min. Sequencing was conducted by Beijing Tianyi Huiyuan Biotech Co., Ltd. New sequences are deposited in GenBank (Table 2).

**Table 2.** Molecular samples included in this study.

Taxa	Museum voucher	Code	Locality	locus					
				12S	16S	cytb	COI	ND4	Reference
*Gloydiuslateralis* sp. nov.	CIB 119377 (JZ02)**	CIB	JNNR	ON362225	ON362229	ON423417	ON399075	ON423421	This study
*G.lateralis* sp. nov.	IVPP OV 2727 (JZ01)*	IVPP	JNNR	ON362226	ON362230	ON423418	ON399076	ON423422	This study
*G.lateralis* sp. nov.	CIB 119378 (JZ03)*	CIB	JNNR	ON362227	ON362231	ON423419	ON399077	ON423423	This study
*G.lateralis* sp. nov.	CIB 119379 (JZ04)*	CIB	JNNR	ON362228	ON362232	ON423420	ON399078	ON423424	This study
*G.angusticeps*.	IOZ 002317*	G1	Golog, Qinghai	KY040541	KY040572	KY040627	KY040604	KY040647	Shi et al. 2018
*G.angusticeps*	IVPP OV 2634**	G5	Zoige, Sichuan	KY040545	KY040577	KY040631	KY040609	KY040652	Shi et al. 2018
*G.blomhoffii*	B524	B524	Japan	AY352719	AY352719	AY352751	–	AY352814	Malhotra and Thorpe 2004
*G.brevicaudus*	DL70	B1	Liaoning	KY040552	KY040584	HQ528467	–	HQ528303	Shi et al. 2017
*G.caraganus*	CR1	CR1	Kazakhstan	–	–	MF490455	–	MF490453	Shi et al. 2017
*G.caraganus*	RIZ 20426.1	426	Kyzylorda, Kazakhstan	MZ958021	MZ957012	MZ959165	–	MZ959158	Shi et al. 2021
*G.caucasicus*	RIZ 29913	913	Mazandaran, Iran	MZ958022	MZ957013	MZ959166	–	MZ959159	Shi et al. 2021
*G.caucasicus*	NEZMUT_61	NE61	Alborz, Iran	–	–	MH378692	–	MH378729	Asadi et al. 2019
*G.changdaoensis*	SYNUSHF01△	C1	Changdao, Shandong	KY040522	KY040554	KX063823	KY040586	KX063796	Shi et al. 2017
*G.cognatus*	CIB-QY224	QY224	Zoige, Sichuan	KY040529	KY040561	KY040619	KY040593	KY040640	Shi et al. 2017
*G.cognatus*	SYNU 13109I3	I3	Saihan, Inner Mongolia	KY040531	KY040563	KY040621	KY040595	KY040642	Shi et al. 2017
*G.halyshalys*	SYNU 1510151	H9	Greater-Xing’an, Heilongjiang	KY040528	KY040560	KY040618	–	KY040639	Shi et al. 2017
*G.huangi*	CIB 533422012	MK	Mangkang, Tibet	–	MZ957017	MZ355578	–	MZ355578	Shi et al. 2022
*G.huangi*	KIZ 027654*	027654	Chaya, Chamdo, Tibet	MK227409	MK227412	MK227415	–	MK227418	Wang et al. 2019
*G.intermedius*	SYNU 150622	22	Zhuanghe, Liaoning	KY040524	KY040556	KY040617	–	KY040638	Shi et al. 2017
*G.liupanensis*	GP198	S083	Ningxia	–	MK193903	MK201255	–	JQ687472	Li et al. 2019; Xu et al. 2012
*G.lipipengi*	IVPP OV 2720	G2	Zawalong, Zayu, Tibet	KY040542	KY040574	KY040628	AY352751	KY040649	Shi et al. 2021
*G.liupanensis*	LP1	LP1	Guyuan, Ningxia	MZ958024	MZ957015	MZ959168	KY040599	MZ959161	Shi et al. 2021
*G.liupanensis*	LP4	LP4	Guyuan, Ningxia	MZ958025	MZ957016	MZ959169	ON399079	MZ959162	Shi et al. 2021
*G.liupanensis*	TC1	TC1	Tanchang, Gansu	MZ958023	MZ9570124	MZ959167	ON399080	MZ959160	Shi et al. 2021
*G.monticola*	SYNU 1607DL1	DL1	Dali, Yunnan	KY040549	KY040581	KY040635	–	MG025935	Shi et al. 2017
*G.qinlingensis*	SYNU QL1△	QLS	Xunyangba, Shanxi	KY040534	KY040566	KY040623	KY040598	KY040644	Shi et al. 2017
*G.rickmersi*	MHNG 2752.69	R1	Kyrgyzstan	–	–	–	–	KM078592	Wagner et al. 2016
*G.rubromaculatus*	IOZ 032317**	Y2	Qumarleb, Qinghai	KY040546	KY040578	KY040632	KY040610	KY040653	Shi et al. 2017
*G.stejnegeri*	SYNU 1508S4△	S4	Linfen, Shanxi	KY040537	KY040569	KX063818	KY040601	KX063791	Shi et al. 2017
*G.shedaoensis*	SYNU 110D2△	D2	Lvshun, Liaoning	KY040523	KY040555	KX063819	KY040587	KX063792	Shi et al. 2017
*G.strauchi*	SYNU 1501G3△	G3	Kangting, Sichuan	KY040543	KY040575	KY040629	KY040607	KY040650	Shi et al. 2017
*G.strauchi*	SYNU 1508G4	G4	Litang, Sichuan	KY040544	KY040576	KY040630	KY040608	KY040651	Shi et al. 2017
*G.swild*	IVPP OV 2725	GR1	Heishui, Aba, Sichuan	OK210582	OK184551	OK239647	–	OK239652	Shi et al. 2021
*G.swild*	IVPP OV 2726	GR2	Heishui, Aba, Sichuan	OK210583	OK184552	OK239648	–	OK239653	Shi et al. 2021
*G.tsushimaensis*	–	Ts1	Japan	JN870203	JN870196	JN870203	JN870203	JN870211	Fenwick 2011
*G.ussuriensis*	U1	U1	Heilongjiang	KP262412	KP262412	KP262412	KP262412	KP262412	Xu et al. 2012
*Deinagkistrodonacutus*	–	A	Fujian	DQ343647	DQ343647	DQ343647	DQ343647	DQ343647	Yan et al. 2008

Note: **, holotype; *, paratype; △, topotype. The missing data are marked as “–”.

For phylogenetic comparisons, corresponding sequences of 22 recognized species of the genus *Gloydius*, and one representative of the outgroup (*Deinagkistrodonacutus*) were obtained from GenBank (Table 2; Shi et al. 2021). Sequences were assembled, and aligned using MEGA 6 (Tamura et al. 2013) with default settings, and were further revised manually when necessary. With respect to the different evolutionary characters of each molecular marker, the dataset was initially split into eight partitions by gene and codon positions and then combined into nine partitions taking advantage of PartitionFinder 2.1.1 (Lanfear et al. 2012) to find similarly evolving partitions.

A Bayesian phylogenetic analysis was performed using MrBayes 3.1.2 (Ronquist et al. 2012). All searches consisted of three heated chains and a single cold chain. Three independent iterations each comprising two runs of 100 million generations were performed, sampling every 10,000 generations, and parameter estimates were plotted against generation. The first 25% of the samples were discarded as burn-in, resulting in a potential scale reduction factor (PSRF) of < 0.005. A maximum-likelihood analysis was run with the IQtree tool in the webserver CIPRES (https://www.phylo.org/index.php), with 1000 fast bootstrap repeats. The General time-reversible (GTR) model, the most probable substitution model for the corrected ND4*p*-distance matrix was calculated in PAUP 4.0 (https://people.sc.fsu.edu/~dswofford/paup_test/).

## ﻿Results

### ﻿Morphology

**Figure 2.**
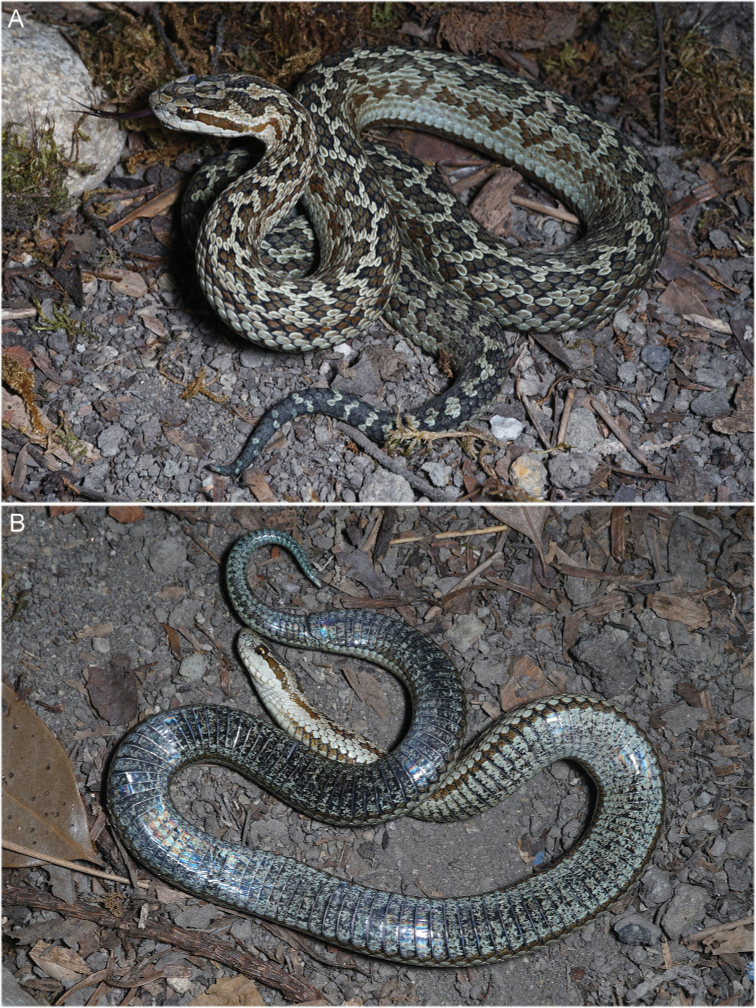
Holotype of *Gloydiuslateralis* sp. nov. in life, adult female, CIB 119377 (JZ02) **A** dorsolateral view **B** ventral view.

**Figure 3.**
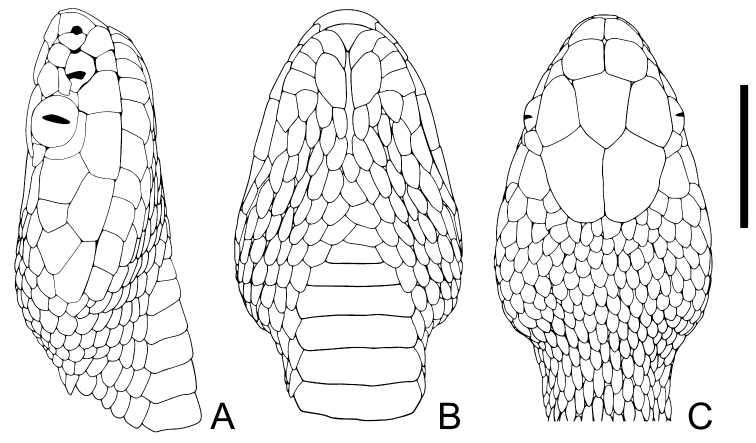
Head squamation of holotype of *Gloydiuslateralis* sp. nov. CIB 119377 (JZ02) **A** lateral view **B** ventral view **C** dorsal view. Scale bar: 10 mm.

**Figure 4.**
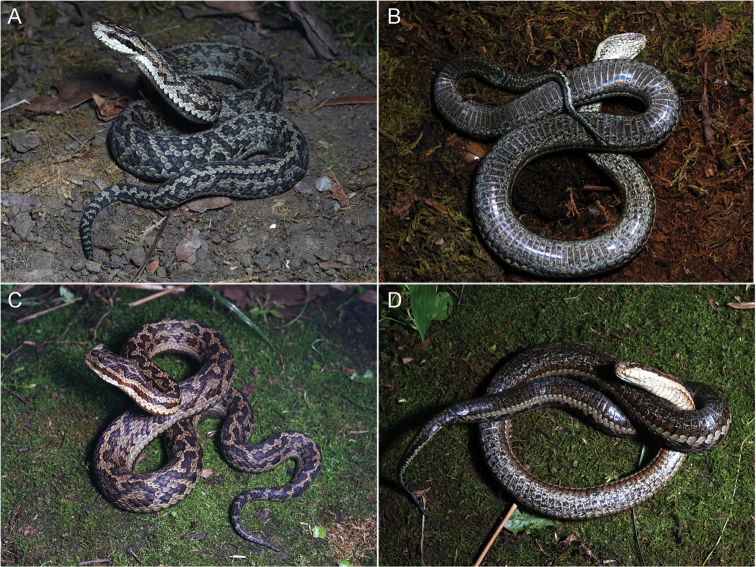
Paratypes of *Gloydiuslateralis* sp. nov. in life **A, B**CIB 119379 (JZ04), adult female in dorsolateral and ventral views **C, D** CIB119378 (JZ03), adult female in dorsolateral and ventral views.

**Figure 5.**
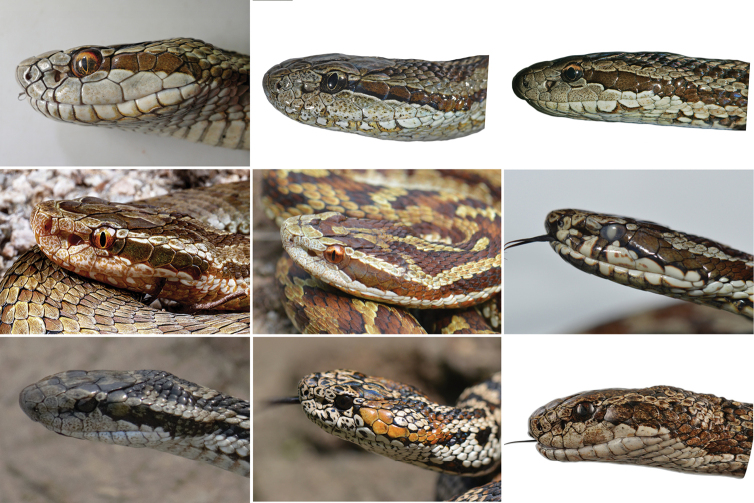
Lateral (dorsolateral) heads of the alpine pitvipers (*Gloydius*) distributed in Sichuan and Tibet (not to scales) **A***G.lateralis* sp. nov., CIB 119377 (JZ02), female, from Jiuzhaigou National Nature Reserve, Sichuan **B***G.swild*, IVPP OV 2725, female, from Heishui, Sichuan **C***G.angusticeps*, IVPP OV 2634, male, from Zoige, Sichuan **D***G.liupanensis*, male, from Longnan, Gansu **E***G.qinlingensis*, male, from Ningshaan, Shaanxi **F***G.strauchi*, SYNU1508G4, male, from Litang, Sichuan **G***G.lipipengi*, IVPP OV 2720, male, from Zayu, Tibet **H***G.rubromaculatus*, male, from Yushu, Qinghai (not included in this study) **I***G.huangi*, CIB 533422012, male, from Hola, Mangkang. Copyright: Sheng-Chao Shi (**A, I**), Jing-Song Shi (**B, C, E, F, G, H**), Zu-Yao Xia (**D**).

**Figure 6.**
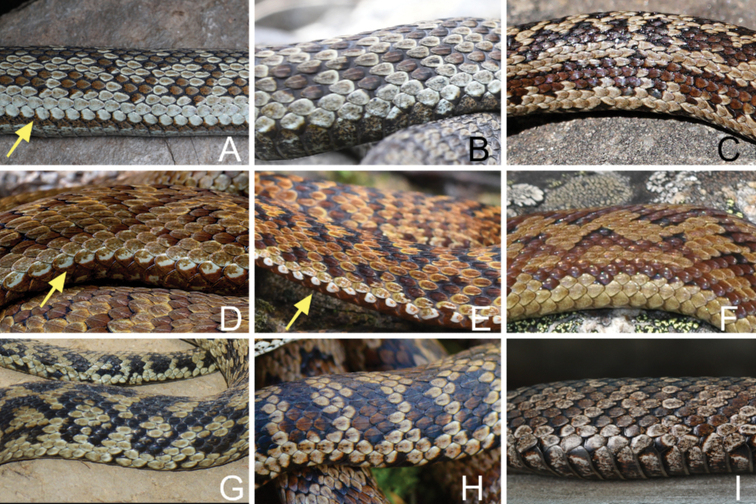
Lateral (dorsolateral) view of the alpine pitvipers (*Gloydiusstrauchi* complex) that distributed in Sichuan and Tibet (not to scales); the lateral stripes are pointed by arrows **A***G.lateralis* sp. nov., CIB 119377 (JZ02), female, from Jiuzhaigou, Sichuan **B***G.swild*, IVPP OV 2725, female, from Heishui, Sichuan **C***G.angusticeps*, male, from Golog, Qinghai (not included in this study) **D***G.liupanensis*, male, from Longnan, Gansu **E***G.qinlingensis*, male, from Ningshaan, Shaanxi **F***G.strauchi*, from Sichuan **G***G.lipipengi*, IVPP OV 2720, male, from Zayu, Tibet **H***G.rubromaculatus*, male from Yushu, Qinghai **I***G.huangi*, CIB 533422012, male, from Hola, Mangkang. Copyright: Sheng-Chao Shi (**A, I**), Jing-Song Shi (**B, E, G, H**), Hong Zhao (**C**), Zu-Yao Xia (**D**) and Zhi-Yuan Tang (**F**).

Comparative data of specimens examined are listed in Tables 1, 2, and 4, as well as Figs 2–6. The holotype and paratypes are illustrated in Figs 2–4. The *Gloydius* specimens from JNNR are different from recognized species of the genus by a combination of morphological characters including ventrals 151–163 (*n* = 9), subcaudals 38–49, laurel green dorsal body with deep-colored patches, and regular greyish brown ventrolateral stripes.

### ﻿Molecular phylogeny

**Figure 7.**
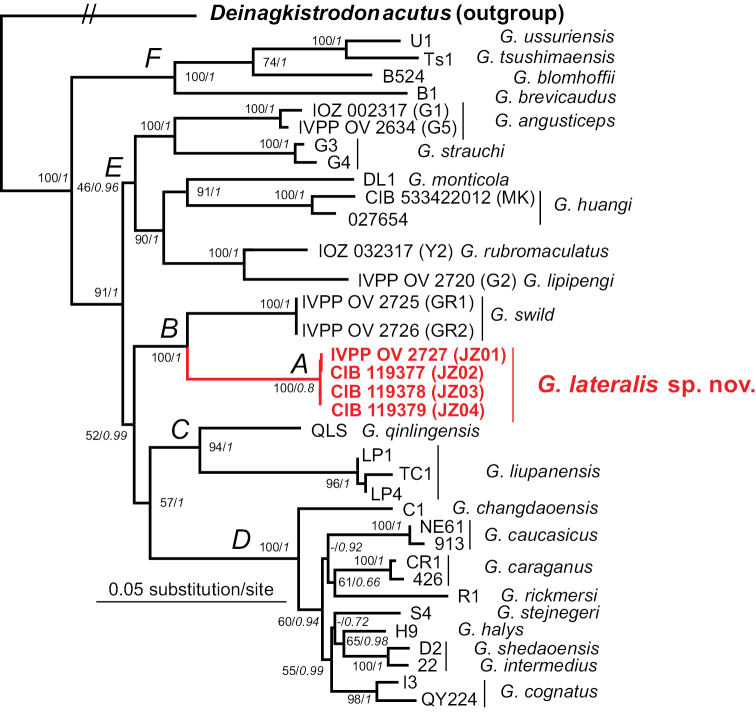
Maximum-likelihood tree of the genus *Gloydius* based on 12S, 16S, COI, ND4 and cytb sequences, with the maximum likelihood bootstrap supports (left, regular) and Bayesian posterior probabilities (right, italic) displayed on the nodes (those < 50% are displayed as “-”).

The final dataset with 3,722 bp of 37 specimens was analysed in this study. The evolutionary models assigned to each of the nine partitions by PartitionFinder are shown in Table 3. In this study, the topological structures of the maximum likelihood (ML) and Bayesian inference (BI) trees are generally consistent (Fig. 7). The four specimens from JNNR formed a strongly supported monophyletic group. This lineage is sister to the clade of *G.swild* from Heishui, Sichuan. The clade including the new species and *G.swild* is sister to the clade formed by 11 species of the *G.intermedius-halys* complex. The result conforms to the earlier studies of the genus (Xu et al. 2012; Shi et al. 2017, 2018; Wang et al. 2019).

**Table 3.** Partitions and their evolutionary models selected by PartitionFinder 2.1.1.

Partitions	Locus	Length (bp)	Models
Partition 1	*12S*	859	GTR+I+G
Partition 2	*16S*	475	GTR+I+G
Partition 3	cytb pos1, ND4 pos1	577	TVM+I+G
Partition 4	ND4 pos2	222	TVM+I+G
Partition 5	ND4 pos3 and cytb pos3	577	GTR+G
Partition 6	cytb pos2	355	K81uf+I+G
Partition 7	COI pos1	219	GTR+G
Partition 8	COI pos2	219	K81UF+I
Partition 9	COI pos3	219	GTR+I+G

GTR: General Time-Reversible model; K81uf: Kimura 1981 TVM: transversional substitution model; TIM: transitional substitution model; +G: rate heterogeneity; +I: proportion of invariable sites

The corrected *p*-distance based on the ND4 gene between the new species and its closest related congeners, *G.swild* is 6.1%, higher than many pairs of substantial species, such as *G.intermedius* vs *G.shedaoensis* (1.1%), *G.halys* vs *G.cognatus* (3.3%), *G.qinlingensis* vs *G.liupanensis* (3.9%), and *G.lipipengi* vs *G.rubromaculatus* (4.2%; Table 4). Thus, the molecular phylogeny supports these new specimens from JNNR as phylogenetically independent species.

**Table 4.** Corrected distance between *Gloydiuslateralis* and other *Gloydius* species based on ND4 and GTR model.

		1	2	3	4	5	6	7	8	9	10	11	12	13	14	15	16	17	18	19	20	21
1	*G.intermedius* (22)	-																				
2	*G.shedaoensis* (D2)	0.011	-																			
3	*G.halys* (H9)	0.041	0.042	-																		
4	*G.cognatus* (I3)	0.033	0.033	0.033	-																	
5	*G.stejnegeri* (S4)	0.045	0.050	0.047	0.041	-																
6	*G.rickmersi* (R1)	0.052	0.051	0.054	0.049	0.065	-															
7	*G.caraganus* (CR1)	0.038	0.046	0.049	0.042	0.059	0.050	-														
8	*G.changdaoensis* (C1)	0.054	0.049	0.050	0.042	0.069	0.066	0.054	-													
9	*G.qinlingensis* (QL1)	0.110	0.122	0.106	0.104	0.113	0.113	0.114	0.113	-												
10	*G.liupanensis* (LP1)	0.090	0.099	0.088	0.086	0.097	0.102	0.097	0.095	0.039	-											
11	*G.strauchi* (G3A)	0.098	0.110	0.102	0.097	0.111	0.116	0.107	0.105	0.074	0.059	-										
12	*G.angusticeps* (G5C)	0.101	0.112	0.097	0.099	0.112	0.104	0.102	0.104	0.063	0.068	0.067	-									
13	*G.monticola* (DL1)	0.120	0.130	0.122	0.112	0.137	0.134	0.135	0.120	0.076	0.078	0.079	0.076	-								
14	*G.huangi* (R86*)	0.111	0.123	0.117	0.112	0.122	0.122	0.119	0.124	0.081	0.080	0.086	0.080	0.078	-							
15	*G.rubromaculatus* (Y2*)	0.106	0.115	0.100	0.109	0.118	0.109	0.113	0.112	0.086	0.085	0.090	0.079	0.089	0.085	-						
16	*G.lipipengi* (G2)	0.112	0.125	0.114	0.127	0.124	0.121	0.119	0.132	0.078	0.081	0.092	0.081	0.088	0.089	0.042	-					
17	*G.swild* (GR1)	0.116	0.125	0.110	0.108	0.102	0.123	0.114	0.119	0.099	0.085	0.089	0.086	0.089	0.103	0.085	0.097	-				
18	*G.brevicaudus* (B1)	0.143	0.152	0.135	0.145	0.155	0.150	0.145	0.156	0.117	0.113	0.124	0.121	0.122	0.138	0.124	0.126	0.138	-			
19	*G.ussuriensis* (U1)	0.106	0.116	0.131	0.115	0.133	0.133	0.119	0.123	0.119	0.109	0.110	0.102	0.122	0.105	0.110	0.128	0.118	0.107	-		
20	*G.blomhoffii* (B524)	0.132	0.144	0.148	0.142	0.157	0.147	0.135	0.144	0.119	0.110	0.117	0.110	0.112	0.119	0.116	0.125	0.125	0.117	0.068	-	
21	*G.sushimaensis*_Ts1	0.121	0.135	0.145	0.133	0.152	0.142	0.139	0.146	0.126	0.113	0.118	0.108	0.110	0.121	0.130	0.138	0.133	0.122	0.054	0.053	-
22	*G.lateralis* sp. nov.	0.108	0.112	0.100	0.101	0.111	0.120	0.099	0.100	0.095	0.083	0.090	0.092	0.099	0.101	0.091	0.101	0.061	0.147	0.126	0.142	0.149

Both morphological and molecular analyses support that the specimens from JNNR represent a new species, and it is described herein.

### ﻿Taxonomic account

#### ﻿ Viperidae

Taxon classificationAnimaliaSquamataViperidae

Oppel, 1811

646559CB-25AF-52DD-8FE1-3A68BD6DEC75

Gloydius Hoge & Romano-Hoge, 1981

#### ﻿ Gloydius lateralis

Taxon classificationAnimaliaSquamataViperidae

Zhang, Shi, Jiang & Shi sp. nov.

774B80B3-FCF2-5623-B361-E6D8488FC62C

http://zoobank.org/6553A5A1-6A2A-4605-B5F7-64724A2DAB7B

Figs 2 , 3 , 4 , 5 , 6

##### Chresonymy.

*Gloydiusstrauchi* – Li et al. 2004

##### Holotype.

CIB 119377 (collection number: JZ02, Figs 2–3), аdult female, collected from Zharu Valley, Jiuzhaigou National Nature Reserve, Aba Tibetan and Qiang Autonomous Prefecture, Sichuan Province, China (33.26°N, 103.93°E, 2072 m a.s.l.), leg. Chun-Lin Zhao, Peng Yan, and Tao Yang, 8 Jun. 2021.

##### Paratypes.

Three adult females: IVPP OV 2727, CIB 119378, and CIB 119379 (JZ01, JZ03, and JZ04; Fig. 4), leg. Peng Yan and Mei-Hua Zhang 31 Aug. 2021. Four adult females (SAFS2021001–SAFS2021004), leg. Ping Wang, Jun. 2021; one adult male (CIB 87280), leg. Cheng Li, 23 May 2002. All paratypes were collected from the same locality of the holotype.

##### Diagnoses.

The above-mentioned specimens were identified as members of the genus *Gloydius* based on the small body size, bilateral pits, and divided subcaudal scales (Zhao 2006; Shi et al. 2016, 2017, 2018, 2021). *G.lateralis* sp. nov. differs from other congeneric species by a combination of the following characteristics: (1) relatively larger eyes (ED/HL: 0.145–0.171, *n* = 5); (2) three palatine teeth; (3) 20 or 21 rows of mid-body dorsal scales; (4) ventrals 151–163 (*n* = 9); (5) subcaudals 38–49 (*n* = 6); (6) dorsal body laurel green or light brown with four rows of zigzag, dark brown patches, the medial two rows separated from each other by alternate phyllotaxis pattern in background color; (7) continuous, regular greyish-brown ventrolateral stripe on each side of body and tail.

##### Comparisons.

Compared to other species in the genus *Gloydius*, *G.lateralis* sp. nov. has continuous, regular greyish-brown and pale, greyish-white ventrolateral stripes on both sides (vs disconnected white upper bordered ventrolateral stripes in *G.qinlingensis* and *G.liupanensis*; lacking the ventrolateral stripes in other congeneric species), and relatively larger eyes than the congeneric species (the ratio between the eye diameter and head length ranges from 0.145–0.171 in *G.lateralis* sp. nov. vs < 0.134 in others).

*Gloydiuslateralis* sp. nov. can be differentiated from the species in the *G.blomhoffii* group by having three palatine teeth (vs four), from the *G.halys* complex by having 20 or 21 rows of mid-body dorsal scales (vs 22 or 23).

For species in the *G.strauchi* group, *Gloydiuslateralis* sp. nov. can be differentiated from *G.monticola* by having 20 or 21 rows of mid-body dorsal scales (vs 19 in *G.monticola*). Given the similar to *G.angusticeps*, *G.lateralis* sp. nov. can be differentiated from the latter by the larger eyes (ED/HL 0.145–0.171 vs 0.104–0.119) and the ticker postorbital stripes. Additionally, the ventrolateral stripes sometimes appear in some other *Gloydius* species, such as *G.qinlingensis* and *G.liupanensis*, but *G.lateralis* sp. nov. differs from them by having the ventrolateral stripe lacking a white upper edge. *Gloydiuslateralis* sp. nov. differs from *G.strauchi*, *G.rubromaculatus*, *G.lipipengi*, and *G.huangi* by the triangular head in dorsal view (vs spoon-shaped head: Figs 5, 6).

*Gloydiusswild* is another species from Heishui, Aba, Sichuan (female holotype IVPP OV 2725 and paratype IVPP OV 2726) that is phylogenetically most closely related to *G.lateralis* sp. nov., but *G.swild* can be separated from the latter by significant branch lengths and *p*-distance (6.1%). *G.lateralis* sp. nov. differs from *G.swild* by having fewer ventrals 151–163 (*n* = 9) (vs 168–170, *n* = 2), laurel-green dorsal body with deep-colored patches (vs dark, greyish-brown background dorsal color), relatively larger eyes (ED/HL 14.5–17.1%, *n* = 5 vs 11.5–13.4%, *n* = 2), the thicker postorbital stripes (2/3 the width of the anterior temporal vs half the width of the anterior temporal), and the regular greyish-brown ventrolateral stripes (vs irregular ventrolateral stripes).

##### Description of the holotype.

CIB 119377 (JZ02), adult female, body slender, medium-sized, tail short (SVL 440.0 mm, TL 58.0 mm, TL/TTL 0.116). Head triangular in dorsal view, 1.45× the length of the width, distinct from the neck (HW 15.1 mm, HL 21.9 mm, HH 8.3 mm); snout bluntly protruding (SL 6.4 mm) from dorsal view; upper jaw slightly protruding beyond lower jaw; rostral scales barely seem from dorsal view; canthus rostralis blunt; eyes relatively large (ED 3.2 mm), pupil vertical, ED/HL 0. 146. Pupil vertical. Fang not exceeding third infralabial (Fig. 2).

***Scalation*.** Internals wider than long, near right triangular (IN 4.4 mm); prefrontals larger, pentagonal; frontal shield-like; the curve edges of two parietals contacting 13 small scales posterior to frontal and supraoculars; supraocular large, slightly smaller than frontal (IOS 7.8 mm); remaining dorsal head scales smaller posteriorly, first few rows irregular and smooth, gradually rhomboidal and keeled posteriorly. Nasals partially divided into two parts by two disconnected vertical sutures touching rear edge of nostril; two loreals, upper loreal forms part of canthus rostralis, lower loreals distinctly smaller and join pit; preoculars 3/2 (left/right), upmost forms part of canthus rostralis, lower join the pit; postoculars 2/2, upper pair small, lower pair larger, and crescent-shaped, surrounding about one-third of eye, touching third supralabial; temporals 2+3/2+2. Supralabials 6/6: first supralabial in contact with both parts of nasals; second supralabial smallest, fourth and fifth supralabials longest; third supralabial reaching the bottom of orbit; fourth supralabial slightly larger than the following. Infralabials 10/10; first pair extends behind mental, first four pairs narrow and touching chin shields, fifth and sixth infralabials largest, similar in size; one pair of chin shields enlarged, forming a distinct mental groove. Dorsal body scales rhombic with matte surface, keeled except the rows bordering ventrals, increasing in size from medial to lateral; dorsal scales rows 20-20-15; ventral scales 158; anal undivided; subcaudal scales 45 pairs (Fig. 3).

**Coloration in life (Figs 2, 4–6).** Description based on observation immediately after shedding. Dorsal head gray with distinct smoky-black markings resembling a human in half squat; one gray patch present on middle of frontal; one gray U-shaped marking present on parietals. Lateral head light gray; postorbital stripe otter brown, wider than half of the largest anterior temporal, extending to lateral neck, without white margins; supralabials and infralabials light gray without conspicuous spots; iris bicolored, upper one-third gold, lower part marbled with smoky black; edges of pupil gold. Ventral head white; one faint yellow-orange stripe present on inner edges of infralabials and adjacent edges of contacting scales on both lateral sides of the lower lip. Tone uniformly purple-taupe.

Dorsal body laurel green; two rows of pine-needle colored irregular patches present on dorsolateral body behind head markings, each patch involving several scales (mostly 4–8) on seventh to higher dorsal scale rows, and partially connected or separated by one laurel-green scale; vertebral scales mostly laurel green, forming an alternate phyllotaxis pattern on the body after neck; a row of copper patches present on both sides of lateral body behind postorbital stripe, involving several scales (5–7) on dorsal scale rows 3–6, also partially connected or separated by one laurel-green scale. Ventral body white right behind head, mottled with sparse smoky-black spots, gradually dense to posterior; a distinct, continuous, regular, greyish-brown ventrolateral stripe present on each side of body, behind faint yellow-orange stripe, lie on junction of ventrals and lower edge of first dorsal scales. Dorsal tail smoky black, covered with a dozen of small, laurel-green patches or transverse bands. Ventral tail laurel green with dense, smoky-black spots, continuous, regular, greyish brown, extending from body to middle of ventrolateral tail. Skin between all dorsal scales black. Front edge of most dorsal scales dyed black.

##### Variations.

Measurements and body scalation variations are listed in Table 1. One of the paratypes, CIB 119378 (JZ03) has a more deeply brown background color than other specimens, dorsal scales on body ginger, and lateral patches on body chestnut; ventrolateral stripes saddle brown. A small, dark-brown spot presents on the middle of the posterior head of one of the paratypes CIB 119378 (JZ03), IVPP OV 2727 (JZ01). Postoculars 3/3 in CIB 87280, IVPP OV 2727 (JZ01); 2/3 in CIB 119379 (JZ04).

##### Etymology.

The specific epithet *lateralis* refers to the unique continuous, regular, greyish-brown ventrolateral stripes at the junction of ventrals and the first row of dorsal scales. The common name is suggested as “Jiuzhai pitviper” in English, “Jiǔ Zhài Fù” (九寨蝮) in Chinese, refer to its type locality, JNNR.

##### Distribution and ecology.

**Figure 8.**
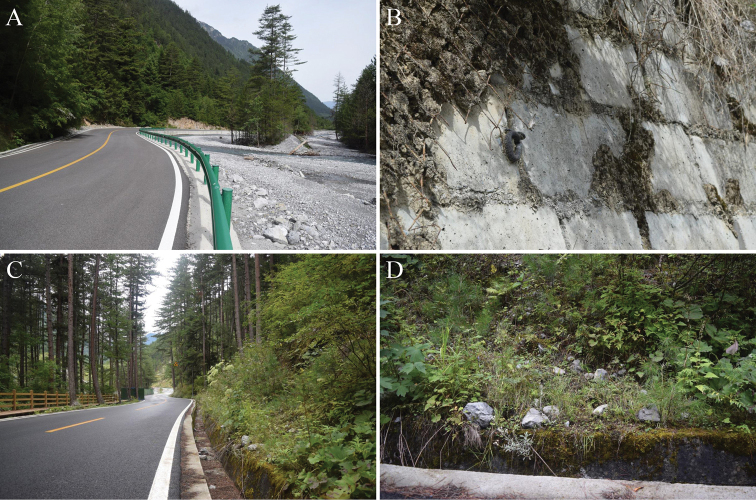
**A, C** Macrohabitats and **D** microhabitats of *Gloydiuslateralis* sp. nov. at the Jiuzhaigou National Nature Reserve. Note that another *Protobothropsjerdonii* sympatric to *G.lateralis* sp. nov. is shown in **B**.

At present, *G.lateralis* sp. nov. is only known from JNNR, Sichuan, China. The type specimens were collected from the middle of June to the end of August. *Gloydiuslateralis* sp. nov. is active on sunny days by the roadside in a hot, dry valley (Fig. 8). This species is sympatric with *Protobothropsjerdonii*, *Rhabdophisnuchalis*, and *Scincellatsinlingensis*. The food spectrum of the new species includes small mammals based on a small patch of fur observed in feces. They fed on suckling mice in captivity.

## ﻿Discussion

Recent studies continue to improve our understandings of the taxonomy and phylogeny of Asian pitvipers (Xu et al. 2012; Shi et al. 2016, 2017, 2018, 2021; Wang et al. 2019). However, the recent molecular phylogenic trees of Asian pitvipers did not resolve the phylogenic relationship between *Gloydiusqinlingensis* and *G.liupanensis* due to the inconsistencies between ML and BI trees. In this study conversely, the topological structures of the ML and BI trees based on five mitochondrial genome fragments are generally consistent. The clades of *G.qinlingensis* and *G.liupanensis* form a strongly supported monophyletic group. Furthermore, the molecular phylogeny in this study reveals the sister relationship between *Gloydiuslateralis* sp. nov. and *G.swild*, another recently described species from Heishui, Aba, Sichuan (6.1% corrected *p*-distance for ND4; Shi et al. 2021). The linear distance between the type localities of *G.lateralis* sp. nov. and *G.swild* is only 148 km (Fig. 1).

The discovery of *G.lateralis* sp. nov. provides new insights into the diversity and the distribution patterns of Asian pitvipers. The genetic differentiation from its closest congener, *G.swild*, might suggest that the formation of the Qinghai-Tibet Plateau might be one of the key factors to the geographical isolation of the alpine pitvipers in southwest China. As discussed by Shi et al. (2021) and authors of many similar studies (Shi et al. 2017, 2018; Wang et al. 2019), the wide-ranging *G.strauchi* complex spans several biogeographic barriers and distinct environments across poorly investigated regions. The discovery of *G.lateralis* sp. nov. verifies the hypothesis that there might be additional hidden species within the *G.strauchi* complex.

The type locality of *G.lateralis* is located in Jiuzhaigou National Nature Reserve, a world-famous heritage site that receives millions of tourists every year. The only known habitat of the new species is Zharu Valley, and it is now under touristic development. Walkways for tourists have been built in the region, but some people are still venturing off of the walkways. Thus, warning signs are still needed to remind visitors to watch out for the venomous pitviper, since this species and the sympatric *Protobothropsjerdonii* are often found in grass or bushes on both sides of roads. On the other hand, reptiles are one of the vertebrate groups most affected by roads through vehicle collisions, both because they are intentionally killed by drivers, and due to their biological needs, such as thermoregulation, making them more prone to collisions (Gonçalves et al. 2018). The observation of the dead bodies of *G.laterialis* shows the necessity to remind the drivers to slow down, and avoid road killings.

## Supplementary Material

XML Treatment for Viperidae

XML Treatment for Gloydius lateralis
